# The prevalence, incidence and natural history of primary sclerosing cholangitis in an ethnically diverse population

**DOI:** 10.1186/1471-230X-11-83

**Published:** 2011-07-18

**Authors:** Elaine Toy, Sripriya Balasubramanian, Carlo Selmi, Chin-Shang Li, Christopher L Bowlus

**Affiliations:** 1 Department of Medicine, University of California Davis Medical Center, Sacramento, CA USA; 2 Division of Gastroenterology, Kaiser Permanente Medical Group, Sacramento, CA USA; 3 Autoimmunity and Metabolism Unit, IRCCS Istituto Clinico Humanitas, Rozzano, Milan, Italy; 4 Division of Rheumatology, Allergy and Clinical Immunology, University of California Davis, Davis, CA USA; 5 Department of Public Health Sciences, Division of Biostatistics, University of California Davis, Davis, CA USA; 6 Division of Gastroenterology and Hepatology, University of California Davis Medical Center, Sacramento, CA USA

**Keywords:** epidemiology, transplantation, cholangiocarcinoma, inflammatory bowel disease

## Abstract

**Background:**

Primary sclerosing cholangitis (PSC) is a rare chronic cholestatic liver disease often associated with inflammatory bowel diseases (IBD). Current epidemiological data are limited to studies of predominantly Caucasian populations. Our aim was to define the epidemiology of PSC in a large, ethnically diverse US population.

**Methods:**

The Northern California Kaiser Permanente (KP) database includes records from over 3 million people and was searched for cases of PSC between January 2000 and October 2006. All identified charts were reviewed for diagnosis confirmation, IBD co-morbidity, and major natural history endpoints.

**Results:**

We identified 169 (101 males) cases fulfilling PSC diagnostic criteria with a mean age at diagnosis of 44 years (range 11-81). The age-adjusted point prevalence was 4.15 per 100,000 on December 31, 2005. The age-adjusted incidence per 100,000 person-years was not significantly greater in men 0.45 (95% CI 0.33 - 0.61) than women 0.37 (95% CI 0.26 - 0.51). IBD was present in 109/169 (64.5%) cases and was significantly more frequent in men than women with PSC (73.3% and 51.5%, respectively, p = 0.005). The cumulative average yearly mortality rate was 1.9%. Age and serum sodium, creatinine and bilirubin at diagnosis and albumin at last entry were identified as significant factors associated with death, liver transplant or cholangiocarcinoma.

**Conclusions:**

The incidence and prevalence of PSC observed in a representative Northern California population are lower compared to previous studies in Caucasian populations and this might reflect differences in the incidence of PSC among various ethnic groups.

## Background

Primary sclerosing cholangitis (PSC) is a chronic cholestatic liver disease of unknown etiology [1] characterized by classic findings of multiple segmental strictures in the intra- and extra-hepatic bile ducts secondary to inflammation and obliterative fibrosis. Less common, the small duct PSC subtype is characterized by a normal cholangiogram and a better prognosis [2]. Inflammatory bowel disease (IBD) is found in 65-90% of PSC cases and though usually classified as ulcerative colitis (UC), the IBD associated with PSC has a unique phenotype including pancolitis, ileitis, rectal sparing and is often asymptomatic [3,4].

Both environmental and genetic risk factors have been associated with PSC susceptibility, in some cases being common to the IBD background, as in the case of smoking [5-7]. Interestingly, the association of PSC with the human leukocyte antigen DR3-B8-A1 haplotype found in Northern European populations [8,9] has not been reproduced in Italian or Brazilian PSC cohorts suggesting that genetic risks for PSC may vary between populations [10,11]. In African-Americans patients listed for liver transplantation in the US, we have recently shown that PSC is associated with HLA-B8, but not HLA-DR3 suggesting a common genetic risk factor near the HLA-B locus [12].

The overall risk and clinical presentation of PSC also appears to vary between populations. Population-based studies in different geographical areas of primarily Northern European ancestry have demonstrated annual incidence rates ranging between 0.41 and 1.2 per 100,000 person-years [13-18]. In populations of Asia, Southern Europe and Alaska, the prevalence of PSC appears much lower and with a lower frequency of IBD [19-25]. In contrast, small case series suggested that IBD patients of African descent are at greater risk of PSC, [26,27] although this has not been recapitulated in a larger study [28]. Our own analysis of liver transplant registrants in the US identified African-American race as a significant risk factor for PSC patients even after controlling for socioeconomic and other factors [12]. The aim of the present study was to determine whether the ethnic differences identified in the liver transplant registrant population extends to PSC patients in general by measuring the incidence, prevalence, natural history, and IBD co-morbidity in a large health maintenance organization consisting of over 3 million subjects representing an ethnically diverse population.

## Methods

### Study population

We utilized the membership database of the northern California Kaiser-Permanente Medical Care Plan (KP). This is a prepaid health maintenance organization that provides medical care to approximately 25-30 percent of the population primarily located in urban areas of the greater San Francisco Bay and Sacramento metropolitan areas. Comparison of KP membership data and census data demonstrate that the KP membership is closely representative of the Northern California population in many demographic variables with the exception of small differences in education and income [29]. A random sample of 5,080, members in 2001 was comprised of 64% non-Hispanic white, 12% Hispanic, 6% African-American, 16% Asian and 3% other [30]. According to 2000 US census data the population of the twelve counties served by KP was 51.1% non-Hispanic white, 19.8% Hispanic, 7.5% African-American, 17.0% Asian and 4.6% other. The study was conducted with the approval of the Kaiser Foundation Research Institute Institutional Review Board.

### Case ascertainment

After approval by the Institutional Review Board, the northern California KP database was searched for PSC diagnoses entered between January 2000 and October 2006. Unlike ICD-9 coding which does not allow for a specific code for PSC, the KP database utilizes a unique code for PSC. The charts of all subjects identified were then reviewed for biochemical, cholangiographic, and/or histological evidence of PSC. Inclusion criteria consisted of radiographic evidence of PSC on cholangiogram or histological evidence on liver biopsy. When neither test was positive for PSC the case was excluded from further analysis despite the PSC coding. Small duct PSC was defined as histological findings consistent with PSC and a normal cholangiogram. Exclusion criteria included secondary causes of biliary sclerosis. Natural history endpoints included liver transplant, cholangiocarcinoma, and death. Additional collected data included age, gender, date of diagnosis, laboratory studies at diagnosis, means of diagnosis, and the presence of IBD. Data on race and ethnicity were recorded when mentioned in the medical record but were available on less than half of the cases and were not included in this analysis.

### Statistical Analysis

Age- and/or gender-adjusted rates and 95 percent confidence intervals were calculated using direct standardization with the age and/or gender distribution of the 2000 US population as the reference population. To compare the female and male groups for the (overall) age-adjusted incidence rates of PSC, we used the method of Dobson et al. (1991) to calculate the confidence interval (CI) for each gender group [31]. We used time to any of three events; death, cholangiocarcinoma, or liver transplant, as a response variable and used the Cox proportional hazards model to investigate what factors had a statistically significant effect on hazard rates. Two-sided Fisher's exact test and Pearson's exact test were used to compare proportions and crude incidence rates, respectively. Wilcoxon-Mann-Whitney test was used to compare continuous variables. All analyses were two-tailed and a p-value of < 0.05 was considered as statistically significant.

## Results

### Patient Characteristics

We identified 169 patients who fulfilled the diagnostic criteria for PSC during the study period (Table 1). Of these 101 (59.8%) were males. The mean age at diagnosis was 44 years (range 11-81). There were 12 (7%) cases diagnosed before the age of 18 years. Age groups at diagnosis in adult cases included 49 (30%) at ages 18-35 years, 82 (50%) at ages 36-65 years, and 21 (13%) at ages >65 years. Five (3%) cases were considered to have small duct PSC based upon a normal appearing cholangiogram and liver histology consistent with PSC.

**Table 1:** Demographic, biochemical, and clinical characteristics of 169 PSC cases diagnosed within a northern Californian HMO in the 2000-2006 period

Age at diagnosis, *years*	44.2 ± 17.4	(11-81)
Males, *n *(%)	101 (59.8)	
Ethnicity,*n *(%)		
White	51 (30.2)	
African American	15 (8.9)	
Hispanic	9 (5.3)	
Asian/Pacific Islander	3 (1.8)	
Other/Unknown	91 (53.8)	
**Laboratory Values at Diagnosis**		
Alkaline Phosphatase, *IU/L*	283.4 ± 256.8	(30 - 1355)
Aspartate Aminotransferase, *IU/L*	66 ± 65.7	(13 - 407)
Alanine Aminotransferase, *IU/L*	70.3 ± 71.9	(11 - 339)
Total bilirubin, *g/dL*	2.3 ± 3.7	(0.2 - 19.7)
Serum albumin, *g/dL*	3.8 ± 0.8	(1.3 - 5.1)
International Normalized Ratio	1.1 ± 0.2	(0.9 - 2.7)
MELD	9.6 + 4.1	(6.4 - 27.4)
IBD, *n *(%)	109 (64.5)	
Ulcerative colitis	95 (56.2%)	
Crohn's disease	13 (7.7%)	
Indeterminate colitis	1 (0.6%)	
Outcomes, *n*		
Liver Transplantation	23	
Cholangiocarcinoma	7	
Death	25	

All continuous variables are expressed as mean ± standard deviation (range).

IBD was present in 109 (64.5%) PSC cases and was classified as UC in 95, CD in 13, and indeterminate colitis in one case. Patients with PSC and IBD were significantly younger with a mean (± standard deviation) age of 41.1 ± 1.6 years compared to 49.4 ± 2.4 years in PSC cases without IBD (P = 0.005). A significant difference in the frequency of IBD by gender was observed with IBD diagnosed significantly more frequently in men compared to women (73.3% and 51.5%, respectively, P = 0.005). No significant differences in age at diagnosis were observed between genders.

### Prevalence and Incidence

In December 2005 there were 140 cases of PSC alive and included in the database. The KP membership at this time consisted of 3,236,094 members giving an overall age-adjusted prevalence of 4.03 per 100,000 (95% CI 3.36 - 4.70). Gender specific age-adjusted prevalence was 4.92 (95% CI 3.86 - 5.97) and 3.19 (95% CI 2.36 - 4.02) for men and women, respectively.

**Figure 1:**
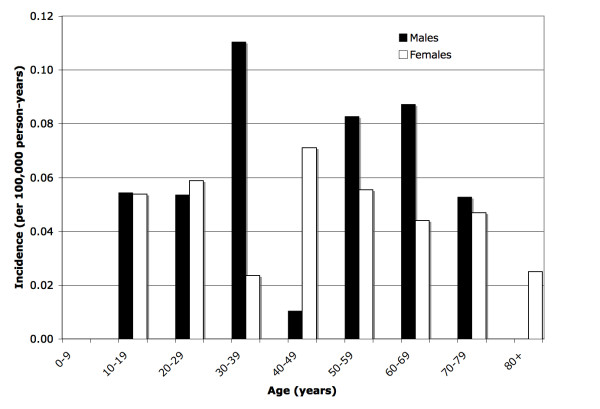
**Adjusted Incidence Rates of Primary Sclerosing Cholangitis in Northern California by Age and Gender, 2000-2006**.

Eighty-one of the 169 cases were incident between January 2000 and October 2006. The overall age-adjusted PSC incidence was 0.41 (95% CI 0.32 to 0.51) per 100,000 person-years. Stratified by gender, the age-adjusted incidence rate for males was numerically greater than females (0.45 (95% CI 0.33 - 0.61) and 0.37 (95% CI 0.26 - 0.51) per 100,000 person-years, respectively). Figure 1 illustrates the adjusted incidence rates by age groups and gender.

### Outcomes

Twenty-three (13%) PSC cases underwent liver transplantation, 9 of which were performed during the study period. Seven (4%) PSC cases were diagnosed with cholangiocarcinoma and 25 (13%) deaths occurred during the study period with 16 being liver-related (Table 2). Regression analyses based on the Cox proportional hazards model demonstrated that age at diagnosis (p = 0.02), serum sodium at diagnosis (p = 0.02), creatinine at diagnosis (p = 0.03), bilirubin at diagnosis (p = 0.003), albumin at diagnosis (p = 0.004), and albumin at last entry (p = 0.005) were statistically significant factors associated with death, liver transplantation or cholangiocarcinoma (Table 3). More specifically, the hazard rate was significantly increased with the increase of age at diagnosis, sodium at diagnosis and creatinine at diagnosis and significantly decreased with the increase of albumin at diagnosis and the increase of albumin at last entry. Race (White versus non-White) was not a significant factor in this analysis.

**Table 2:** Cause of death in PSC patients from 2000-2006

Cause		Number of Deaths
**Liver-related**		
	Liver failure	7
	Hepatorenal syndrome	4
	Cholangiocarcinoma	3
	Liver Cancer, unspecified	1
	Cholangitis/Sepsis	1

**Non Liver-related**		
	Colon Cancer	2
	Myocardial infarction	2
	Squamous Cell Cancer	1
	Pulmonary fibrosis	1
	Pulmonary embolism	1
	Urosepsis	1
	Unknown	1

**Table 3:** Univariate Analysis of Proportional Hazards Regression to Death, Cholangiocarcinoma or Liver Transplant

Characteristic	Hazard ratio (HR)	95% HR confidence interval	P-value
**Gender**	1.065	(0.515, 2.200)	0.87
			
**Race**	0.835	(0.3, 2.327)	0.73
White			
Non-white			
			
**Age at diagnosis**	1.029	(1.004, 1.054)	**0.02**
			
**IBD**	0.657	(0.315, 1.373)	0.26
No			
Yes			
			
**Lab measurement at study entry**			
Sodium	1.144	(1.022, 1.28)	**0.02**
Creatinine	4.034	(1.116, 14.584)	**0.03**
Alkaline phosphatase	1.000	(0.999, 1.002)	0.47
Alanine aminotransferase	0.997	(0.993, 1.001)	0.14
Aspartate aminotransferase	0.996	(0.992, 1.001)	0.13
Bilirubin	1.122	(1.039, 1.211)	**0.003**
INR	1.569	(0.874, 2.818)	0.13
Albumin	1.013	(0.938, 1.095)	0.74
MELD	1.066	(0.998, 1.139)	0.06
			
**Lab measurement at last entry**			
Sodium	0.941	(0.804, 1.101)	0.45
Creatinine	1.873	(0.711, 4.932)	0.20
Alkaline phosphatase	1.001	(0.999, 1.003)	0.51
Alanine aminotransferase	0.999	(0.992, 1.006)	0.76
Aspartate aminotransferase	1.000	(0.994, 1.006)	0.99
Bilirubin	1.09	(0.982, 1.210)	0.11
International normalized ratio	1.335	(0.440, 4.045)	0.61
Albumin	0.329	(0.153, 0.709)	**0.005**

## Discussion

The results of our study, which to our knowledge is the largest population-based case-finding study on PSC, demonstrate a prevalence of 4.03 cases per 100,000 and an incidence of 0.41 per 100,000 person-years. Similar to other populations, the majority of cases are males and IBD, particularly UC, is associated with PSC and is more frequent in males compared to females. We also identified several variables associated with the major disease outcomes, i.e. death, liver transplantation and cholangiocarcinoma.

PSC is a rare cholestatic disorder of unknown etiology for which there is no effective therapy. Although the prevalence of the disease appears to vary widely in different ethnic and racial groups, most population-based studies of PSC have been in relatively homogeneous populations in terms of ethnic and racial composition [13-17]. We sought to determine the prevalence and incidence of PSC in an ethnically and racially diverse population. The Northern California region is comprised of many different ethnic and racial groups with non-Hispanic whites making up only a slight majority of 51.1% based on 2000 US Census data. Because a strong association between PSC and IBD has always been observed to a lesser or greater degree and the risk of IBD varies between ethnic and racial groups, the prevalence of PSC might be similarly affected. Alternatively, the genetic basis of PSC appears to be independent of IBD genetic risk factors suggesting that there may not be a strict correlation between rates of IBD and PSC [9,32].

Similar to other PSC cohorts, we observed a predominance of males with a mean age at diagnosis in the mid-forties. The frequency of IBD was similar to other studies and we identified a significantly lower frequency of IBD in women with PSC, which is consistent with the findings of other smaller studies [33,34]. These rates should be considered a lower limit of the actual frequency. Some subjects did not have a record of a colonoscopy and often times the IBD of PSC is asymptomatic [3,35,36]. Whether the gender differences we observed are due to differences in true IBD prevalence or in rates of diagnosis warrant further investigation.

One of the most striking differences in our study compared to prior studies is the lower incidence and prevalence of PSC in this population compared to populations of Olmstead County, Alberta, Wales and Norway. This would seem to contradict the assertion by some authors that there is an increasing incidence of PSC diagnoses since the widespread use of endoscopic retrograde cholangiography and magnetic resonance cholangiography was introduced. One possible explanation for this discrepancy could be the result of under diagnosis leading to an underestimation of the true disease prevalence. PSC is a rare disease that may not be recognized by a clinician without particular skills or awareness. In addition, because there is not an effective therapy for PSC and liver test abnormalities are frequent in IBD, some clinicians may prefer not to confirm the diagnosis of PSC when it is suspected, particularly if an invasive test is needed. It is notable that our estimates of PSC prevalence and incidence are similar to those of another recent large study of a subset of the British population (total population of 2,027,909), which like the present study, was not based on one or a few hospitals with a particular interest in PSC [17].

Alternatively, this discrepancy might be due to a lower incidence of IBD, particularly UC, within Northern California. However, a recent study of the KP database determined that the incidence and prevalence of UC and CD is similar to or greater than that of Olmstead County, Manitoba and Europe [37]. Nevertheless, differences in the ethnic distribution between IBD and the total population have been noted[37,38]. A prior study of a subset of this population in Oakland, CA reported that non-Hispanic whites made up only 64% of the total population but represented 80% of the IBD cases [38]. In contrast, Hispanics and Asian Americans comprised a disproportionately smaller percentage of the IBD cases compared to the total membership. In the more recent study of the total KP population, African Americans and Asians constituted 16% and 9% of the total IBD population, respectively, compared to 6% and 16% of the membership[37]. Further, other studies suggested that PSC is rare in Asia and that African Americans are at greater risk of developing extra-intestinal manifestation of IBD, including PSC [23,24,26-28]. Ethnicity was documented in the medical record in fewer than half of the cases preventing us from drawing conclusions about the effect of ethnicity on the prevalence or incidence of PSC.

The strengths of our study include the use of comprehensive paper and electronic medical records to ascertain details of the PSC cases, the representative population of the northern California KP and the community care setting as opposed to a tertiary referral center. In contrast to the UK study, we were able to confirm each case of PSC based upon accepted diagnostic criteria. In addition, the size of the population allowed us to more accurately estimate the incidence and prevalence of a relatively rare disease. Therefore, these results are likely to be more representative of the total population.

Our study did have some weaknesses. The lack of ethnic and racial data in a large subset of patients limited our ability to address risk assessment or determination of prognostic factors for the natural course of disease dependent on race. We also note that not all patients with PSC had colonoscopies and therefore our estimates of IBD are likely underestimates of the actual rates. Finally, although this population is generally representative of the total population of Northern California, there is some skewing away from the upper and lower income groups.

## Conclusions

We have reported on the largest population based study of PSC to date and the only one to include an ethnically diverse population. Although the common features of age at diagnosis, male predominance and presence of IBD are similar to other studies, we observed lower PSC prevalence and incidence rates than reported in most other populations. Further studies in this or other similar populations will be helpful in determining if there are significant differences in PSC susceptibility and clinical outcomes between ethnic groups.

## Abbreviations

PSC: primary sclerosing cholangitis; IBD: inflammatory bowel disease; KP: Kaiser Permanente; UC: ulcerative colitis; CI: confidence interval.

## Competing interests

The authors declare that they have no competing interests.

## Authors' contributions

ET participated in the study design, data abstraction and analysis and assisted with the drafting of the manuscript. SB and CS participated in the study design and data analysis. CSL participated in the data analysis. CLB conceived of the study and participated in its design and coordination and drafting of the manuscript. All authors read and approved the final manuscript.

## Pre-publication history

The pre-publication history for this paper can be accessed here:

http://www.biomedcentral.com/1471-230X/11/83/prepub
